# New evidence on financing equity in China's health care reform - A case study on Gansu province, China

**DOI:** 10.1186/1472-6963-12-466

**Published:** 2012-12-18

**Authors:** Mingsheng Chen, Wen Chen, Yuxin Zhao

**Affiliations:** 1 Department of Health Economics, School of Public Health, Fudan University, P.O. Box 187, 138 Yi Xue Yuan Road, Shanghai, 200032, P. R China; 2 National Health Economics Institute, Peking University Health Science Center, P.O.BOX 218, No.38, Xue Yuan Road, Hai Dian District, Beijing, 100083, P.R. China

**Keywords:** Equity, Chinese health care reform, Financing, Kakwani index

## Abstract

**Background:**

In the transition from a planned economy to a market-oriented economy, China’s state funding for health care declined and traditional coverage plans collapsed, leaving China’s poor exposed to potentially ruinous health care costs. In reforming health care for the 21st century, equity in health care financing has become a major policy goal. To assess progress towards this goal, this paper examines the equity characteristics of health care financing in a province of northwestern China, comparing the equity performance between urban and rural areas at two different points in time.

**Methods:**

Analysis of whether health care financing contributions were progressive according to income were made using the Kakwani index for each of the four health care financing channels of general taxes, public and private health insurance, and out-of-pocket payments. Two rounds of surveys were conducted, the first in 2003 (13,619 individuals in 3946 households) and the second in 2008 (12,973 individuals in 3958 households). Household socio-economic, health care payment, and utilization information were recorded in household interviews.

**Results:**

Low-income households have undertaken a larger share of the health care financing burden in recent years, reflected by negative Kakwani indices, which indicate a regressive system. We found that the indices for general taxation were −0.0024 (urban) and −0.0281 (rural) in 2002, and −0.0177 (urban) and −0.0097 (rural) in 2007. Public health insurance presented different financing distributions in urban and rural areas (urban: 0.0742 in 2002, 0.0661 in 2007; rural: –0.0615 in 2002,–0.1436 in 2007.). Out-of-pocket payments were progressive but not equitable. Public health insurance coverage has expanded but financing equity has decreased.

**Conclusions:**

Health care financing policies in China need ongoing reform. Given the inequity of general consumption taxes, elimination of these would improve financing equity considerably. Optimizing benefit packages in public health insurance is as important as expanding coverage, both for health care financing and for utilization management as well. Although they are progressive, out-of-pocket payments are not equitable in China and have the effect of excluding the poor from health care as they cannot afford to pay for medical care and so withdraw from treatment.

## Background

Questions always follow health care reform. Is the reform designed to improve equity? If so, does it work?

To make health care reform sustainable through financial support and acceptable to a majority of the public, policy-makers and researchers prefer that a health care system should be renovated at the starting point. Consequently, financing equity has been set up as one major policy goal in health care reform across a number of countries [1-3]. In a health care system, equitable financing mechanisms play a significant role in promoting health care access and achieving universal coverage of health services, especially for the poor and vulnerable groups [4-7]. It is widely recognized that health care payments should be set equally according to household ability-to-pay (ATP) [8,9]. Therefore, the evaluation of financing equity is a fundamental study for judging whether health care reform is sound or not, for exploring existing flaws in health funding channels, and most importantly, for finding effective countermeasures to improve deficiencies. The distribution of health care financing has been researched both at an international level, based on equity performance comparisons among continents and countries, and at a national level, focused on the variable equity status of unequal socioeconomic groups [10-13]. However, no quantitative evaluation of the distribution of the health care financing burden in the country of China has been conducted. This paper seeks to address this gap by an equity appraisal study of Gansu, one province in China. Moreover, in contrast to earlier studies on health care financing in one particular period, two sets of data for this study were collected from different years. This allows deliberate consideration of not only whether equity of health care financing in a specific year from any panel of data is sound, but whether the health care financing system is making progress in the evolution of the health care reform in China as well.

Influenced by economic reforms since the 1980s, China’s health care system experienced a huge transformation from a planned economic pattern to a market-oriented model. Along with decentralization of financial responsibility for managing health care, state funding for health care declined rapidly. As a result, the proportion of public financing in the health care system decreased while the share of private financing increased [14,15]. In the early 1990s, healthcare insurances collapsed, such as the shrinking of the Government Welfare Insurance Scheme (GWIS) and the Labor Insurance Scheme (LIS) in urban areas, and the breakup of the Cooperative Medical System (CMS) [16,17]. At the same time, because of the above reasons, financing mechanisms came to primarily rely on out-of-pocket payments (OOP) for health care service [18,19]. This transformation greatly changed China’s health care financing structure. Between 1990 and 2002, the percentage of government spending for health dropped from 25.06% to 15.69%; over the same period, the percentage of public health insurance spending dropped from 39.19% to 19.14%. Conversely, the proportion of OOP payments rose sharply from 35.73% to 57.72% [20]. Consequently, heavy reliance on direct payment resulted in financial difficulties in access to health care, and led to a tiered and segmented health financing system, disproportionately providing health care to the poor and marginalized groups. From 1993 to 2003, the proportion of the population who could not afford outpatient medical treatment rose from 32% to 36% in urban areas and from 32% to 39% in rural areas [21]. The average cost of a single hospital admission is more than twice the average annual income of the lowest 20% of the population in China [22]. The aged population in low income groups tended not to use inpatient services [23].

Confronted with such a situation, growing attention has been given to equity, especially after the recommendation of the World Health Report 2000 which implied that China has a most inequitable health care system. The achievement of financing equity has become the health policy thrust in health care innovation. Since 2003, China has been launching health sector reform gradually and implementing a specific policy package. In particular, this package incorporates innovation in health care financing, such as: an increase in government health input, the introduction of new types of health insurance (known as the Urban Resident Basic Medical Insurance Scheme and the New Rural Cooperative Medical Scheme), and an expansion in health insurance coverage for the uninsured. These policies aim to reduce the share of OOP health payments through increasing the proportion of government health spending and health insurance funding [24].

Since then, China has established a multi-level health care financing system step by step for the urban and rural populations, in an attempt to make significant progress in the equitable distribution of health care funding. In Table 1, the percentage of total expenditure on health (TEH) financed from each of the main sources between 2002 and 2007 is presented. Like most health care financing systems, China’s system draws revenues from four sources: general taxes, public health insurance, private insurance and OOP payments.

**Table 1:** The porportions of health care financing amounts in China’s national health account

**Year**	**General taxes (%)**	**Public health insurance (%)**	**Private health insurance (%)**	**OOP (%)**	Other^*^(%)
**2002**	15.69	15.64	2.09	57.72	8.86
**2003**	16.96	14.78	3.68	55.87	8.70
**2004**	17.04	16.31	3.39	53.64	9.63
**2005**	17.93	15.99	3.55	52.21	10.33
**2006**	18.07	17.55	3.83	49.31	11.24
**2007**	22.31	21.28	3.32	44.05	9.05

Data source: China Health Economic Institute.

*Others mainly refers to donation revenue, recurrent expenses of medical-aid, and health administration fees.

### General taxes

Taxation is a stable resource among health care financing channels, especially in developing and underdeveloped countries. In China, the government budget for health is an important part of the financial resources at all levels of government spending, being mainly financed through a variety of tax revenues. No earmarked health taxes exist in China. The revenue mainly comes from direct taxes and indirect taxes. Direct taxes include personal income taxes, corporate taxes, property taxes, and agricultural taxes in China, while indirect taxes include the value-added tax (VAT), excise taxes, and sales taxes. The taxes above are allocated to all forms of qualified individuals, regardless of whether they are the rich or the poor. Tax revenue is turned over as part of the state financial income, a portion of which is dedicated to health care financing system as the government budget on health. Tax financing usually refers to a pay-as-you-go arrangement, where current revenues are used to finance current expenditures. Generally, the rich pay more taxes than the poor in absolute terms. However, it doesn’t mean that tax funding is always the progressive channel that is preferred by the poor. For instance, the payroll tax, sales tax and cigarette tax are regressive in that the tax rate decreases as the amount subject to taxation increases. In other words, a regressive tax imposes a greater burden on the poor than on the rich. On the contrary, a progressive tax implies that the rich bear more burden than the poor, such as with individual income taxes. In China, the amount financed by taxes is rising steadily, from 15.69% in 2002 to 22.31% in 2007.

### Public health insurance

Public health insurance plays a significant role in the health care financing system. Owing to the dual structure of urban–rural areas in China, two national health insurance schemes have been formed separately. These are the urban health insurance called Basic Medical Insurance (BMI) in cities and counties [25], and the rural health insurance known as the New Rural Cooperative Medical Care System (NCMS) in towns and villages.

The Urban Workers Basic Medical Insurance (UWBMI), the initiation of BMI, was officially launched at the end of 1998 and is run by the local government that organizes universal health insurance for urban formal-sector workers. The premium is funded jointly by employers and employees, while the funding amount depends on the individual’s age. Generally, employers provide 6-8% of employees’ salaries for urban workers under the age of 45 and provide 8-10% of the salaries for those aged 45 or above. The employees themselves contribute around 2% of their salaries [26]. Two measures have been adopted in the ongoing health care reform process to update BMI. First, coverage of the population by UWBMI was expanded by almost 14% from 30.4% in 2002 to 44.2% in 2007 [21,27]. In the reform, not only the workers in state-owned and collective enterprises are insured by BMI, but BMI also covers migrant workers and employees working in private enterprises, foreign-invested enterprises, social organizations, private non-enterprise units, etc. Second, a new type of BMI, named the Urban Resident Basic Medical Insurance scheme (URBMI), has been carried out for the 420 million urban residents not covered by UWBMI [28]. Target groups for the new scheme are urban residents such as students, children, the elderly, the non-employed and the disabled. The new urban scheme is financed largely by the family as a unit with appropriate subsidies granted by government [29]. The financing contribution from households accounts for 64% of the total URBMI premium, around 80–120 RMB per capita per annum [30]. All in all, the core of BMI renovation is the expansion of coverage.

The New Rural Cooperative Medical Scheme (NCMS) is a 2003 initiative to rebuild rural health insurance after the collapse of CMS in the 1990s and to overhaul the health care system in villages. NCMS is a voluntary program that covers only those who join; it has maintained a high level of coverage of the population, increasing from 75.20% in 2004 to 86.20% in 2007. NCMS is funded by equal contributions from every enrollee and by increasingly generous subsidies from central and local governments. NCMS operates at the county rather than the commune level and hence has a much larger risk pool. Finally, NCMS focuses in most localities largely on the costs of inpatient care rather than those of basic medical services which include personal and communal preventive interventions. The premium and reimbursement rate grow steadily every year. Generally, the coinsurance has lowered and the medical services covered by NCMS have increased year by year.

### Private health insurance

In China, private health insurance is at the initial stage without a clear direction. Access to commercial health insurance is highly correlated to economic status and personal awareness of the insurance. The insured also need to consider how much to purchase according to their income. In addition, the premiums paid by lower income groups are only slightly lower than those charged to higher income groups. Private health insurance plays a minor role in health care payments, around 3% of the total health care financing amount.

### Out-of-pocket payments (OOP)

OOP payments are those expenditures that individuals must pay directly to the health care provider without any compensation to the patients. As the complementary mechanism to the other financing channels, the OOP payment has been robust since the 1980s in China, to improve the efficiency and quality of the health care system. However, it has become the main source of the Chinese health care financing system (Table 1). With cost escalation in health care and heavy reliance on OOP payments, paying for health care has become a notable cause of impoverishment for households that lack adequate health insurance. More than 35% of urban households and 43% of rural households have difficulty affording health care, go without, or are impoverished by the costs [31]. Although no effort has been spared in aiming to reduce the share of direct payment in health care reform, the OOP payment still accounts for the largest proportion of the total health care financing amount in China. Evidence has shown that two essential outcomes would be created by the high OOP payments: no seeking of medical care because of economic hardship and catastrophic health expenses accumulated if treatment is continued [32].

However, it is hard to evaluate the impact of health care reform and difficult to explore the effect of the changes in health care financing system pre- and post-reform, especially because of the lack of empirical evidence about the actual degree of inequalities associated with the health care financing mechanisms in China. Meanwhile, it would be difficult to find faults in health care financing practice because they come under the auspices of the “hoped-for health care reform”. Moreover, the change and variation in the extent of equalities across years and regions have not been reviewed in previous studies, and this would shed light on the positive or negative effect of health care financing heavily influenced by the measures in the reform. These issues raise questions for policy makers and researchers on how to assess equity in health care reform to prove whether the equity target has been achieved or not.

The paper begins by presenting China’s health care finance within which the Chinese health policies were developed, illuminating the specific measures of health financing implemented by health care reform, and identifying some key issues to be considered in its evaluation. The next section provides the method used in the assessment of health care financing; more specifically, it outlines how empirical results in urban–rural areas and different times were compared. Data about socioeconomic and health status from the national health investigation are then critically analyzed and evaluated. The final section attempts to draw some conclusions in relation to broad lessons from the Chinese experience.

## Methods

### Data

The data for the analysis came from two rounds of household surveys in Gansu province, China. The two rounds were conducted in 2003 and 2008 in the sampling regions, recording the information in 2002 and 2007, respectively. Gansu province, located in the northwest of China, is one of the impoverished provinces with more than 26 million people. Adopting a multi-stage stratified random sampling method, the survey randomly selected 15 cities or counties. In every city or county, 8 communities or villages were selected by economic level and geographic distribution. Then 33 households were randomly selected from the communities or villages. Finally, 3946 households with 13619 individuals in the year 2003 and 3958 households with 12973 individuals in the year 2008 were effectively collected in the survey. Permission was obtained from all individuals who were enrolled in our study after reading through the consent forms. Table 2 presents detailed data about some descriptive and social-economics characteristics in each income quintile.

**Table 2:** Descriptive statistics of sampling data and socioeconomic characteristics by per capita household expenditure quintiles

**Year**	**Income quintiles**	**Nb of families surveyed**		**Nb of individuals surveyed**		**Annual household expenditure**^**a**^		**Insurance rate (%)**	
		**urban**	**rural**	**urban**	**rural**	**urban**	**rural**	**urban**	**rural**
2002	Q_1_	394	394	1243(21.14%)	1609(20.79%)	3495.50(69.38*)	1722.95(38.00)	5.31	7.61
	Q_2_	395	390	1275(21.68%)	1538(19.87%)	6389.32(96.36)	3200.49(43.63)	17.22	8.09
	Q_3_	396	403	1211(20.60%)	1608(20.78%)	9160.73(122.70)	4351.83(53.47)	27.53	6.87
	Q_4_	394	391	1105(18.79%)	1558(20.13%)	12593.67(163.37)	5714.12(73.02)	41.85	12.81
	Q_5_	395	394	1046(17.79%)	1426(18.43%)	28552.80(1464.54)	14375.75(2346.13)	48.47	13.21
	total	1974	1972	5880	7739	12040.99(15839.00)	5871.69(21286.48)	27.07	9.60
2007	Q1	395	395	1217(21.81%)	1567(21.20%)	7017.00(129.81)	3360.84(66.03)	70.67	95.41
	Q2	397	397	1215(21.77%)	1574(21.29%)	11024.56(153.47)	5464.46(74.36)	70.10	92.12
	Q3	396	395	1126(20.18%)	1489(20.14%)	14496.86(220.37)	7058.36(102.88)	71.31	93.28
	Q4	396	398	1031(18.47%)	1474(19.94%)	18998.99(279.67)	9591.37(136.69)	78.47	95.52
	Q5	395	394	992(17.77%)	1288(17.42%)	30941.74(696.17)	15993.42(392.00)	82.28	96.20
	total	1979	1979	5581	7392	16506.67(10922.06)	8288.91(5835.67)	74.18	94.44

Data source: Author’s calculations from the sample of household survey.

^*a*^*All expenditures are presented in CNY.*

^*^Standard error.

The survey contains extensive information about household socio-economic and demographic characteristics, including gross household income, urban–rural classification, number of family members, sex, age, education attainment, and working status of household members, household goods, and consumption. With regard to household expenditure, monthly expenditure on food, clothing, traffic, communication, housing, water, electricity, fuel, education, travel, entertainment, medical care and other expenditures were recorded in the survey. In addition, information on unexpected expenditures in the previous year was also collected. Concerning health care payment, information was computed through two sources of data: one was gathered from the survey above, and the other data were mainly tariffs for taxes and copayments for public health insurance, which were collected from the local statistics yearbook. Specifically, taxes considered in our study include the cigarette tax, alcohol tax, amusement tax, electricity and gas tax, excise on eating, drinking and lodging, and other consumption taxes. These taxes were approximated by applying specific tax rates to the corresponding expenditures. The private health insurance payment was directly obtained from the survey. The inquiry into out-of-pocket payment involved information about health care expenditures on prescription, outpatient and inpatient care paid by individuals during the latest 2 weeks before the household interview. Health care utilization information on outpatient visits and lengths of hospital stays was also collected in the interview. The study was funded by the Ministry of Health, People’s Republic of China, which agreed to the use of these data for academic research.

### Statistical analysis

In our study, the unit of analysis was the household. Gross income and health financing contributions are aggregated to the household level. The value of household expenditure was used as the measurement of living standard. Adjustment is made for the size and age structure of the household through application of an equivalence scale to both ATP and each component of health financing. The scale used is

$$\mathrm{AE}={\left(A+0.5K\right)}^{0.75}$$

where A is the number of adults in the household and K the number of children (0–14 years) [33].

Although there are many different methods to calculate the equity of health care financing, the approach in our study was to employ progressivity to measure the extent of equity. The progressivity of a health care financing system refers to the extent to which health care payments rise as a proportion of a household’s income when the latter rises. Specifically, we use the Kakwani index of progressivity to calculate the extent of the equity of the health care financing system [34-36].

**Figure 1:**
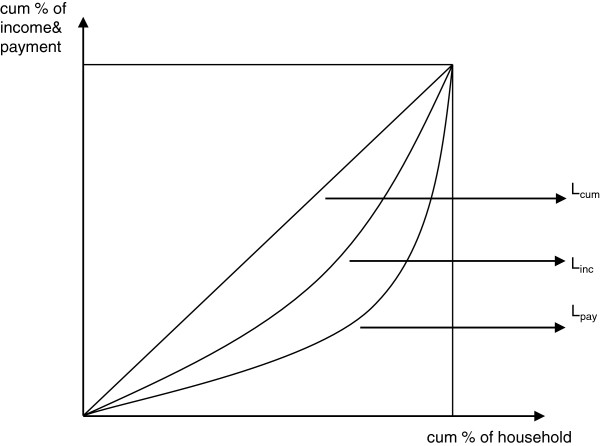
Conceptual cumulative concentration curve for health care payment and income.

Figure 1 displays the conceptual cumulative concentration curve for health care payments and income of household. The Lorenz curve for gross income (L_inc_) represents the relationship between the cumulative percentage of income and the cumulative percentage of the households in population (the households are ranked according to their income, L_cum_), while the concentration curve for health care payments (L_pay_) displays the cumulative healthcare share of payments against the cumulative percentage of the households in population. The Kakwani index of progressivity of health care payments on gross income, is defined by twice the area between the Lorenz curve for gross income (L_inc_) and the concentration curve for health care payments (L_pay_). Thus we have

$$\pi_{k}=2\int_{0}^{1}\left[L_{\mathrm{inc}}-L_{\mathrm{pay}}\right]\mathrm{dp}$$

$$\pi_{k}=2\int_{0}^{1}\left[L_{\mathrm{cum}}-L_{\mathrm{pay}}\right]\mathrm{dp}-2\int_{0}^{1}\left[L_{\mathrm{cum}}-L_{\mathrm{inc}}\right]\mathrm{dp}$$

$$\pi_{k}=C-G$$

where C is the concentration index (CI) of health care payments, defined as the distribution of health care financing contributions across the population ranked by gross income and G refers to the Gini coefficient of gross income. The range of the concentration index is defined by the range (−1, 1). A positive (negative) value of concentration index indicates that the rich (poor) contribute a larger proportion than the poor (rich). The value of the index equals zero if everyone pays the same. However, the value of the concentration index does not denote whether a health care financing system is equitable or not, for it does not take the household income (ATP) into consideration. Only if the proportion of health care payments paid by the rich are not lower than the share of income they received, or the proportion of the payment paid by the poor is not higher than the share of income they received, is the health care system considered to have financing equity. The Kakwani index (π_k_) is calculated by the difference between the concentration index and the Gini coefficient, reflecting the degree of progressivity of health care financing [37]. When the Kakwani index is positive (π_k_ > 0), the health care financing system is progressive, so that the Lorenz curve of income (L_inc_) lies above the concentration curve of payments (L_pay_). When the Kakwani index is negative (π_k_ < 0), the system is regressive, so that the concentration curve of payments (L_pay_) lies above the Lorenz curve of income (L_inc_). When the Kakwani index equals zero (π_k_ = 0), it indicates that the system is proportional, and therefore there is a coincidence of the Lorenz and concentration payments curves. Furthermore, not only can the Kakwani index quantify the degree of financing equity in one certain area in a specific period, but it also enables a comparison of the levels of progressivity at different times and areas without consideration of their different social-economic context for the reason that economic status and health financing factors are included in the model [38]. Thus, the differences of Kakwani index among different regions or years can assess the inequality gaps in areas and variations in times and, consequently, evaluate the financing performance in equity due to different policies in urban–rural areas or interventions at different time intervals.

In addition, a dominance test is added to the progressivity analysis. To establish whether the health care financing reduces inequity, in the sense that lower income individuals contribute a lesser share of financing than the wealthy, compared with their living standards, a test is conducted of whether the concentration curve dominates (lies above) the Lorenz curve of household expenditure. For the dominance tests, standard errors of the ordinates of curves and of differences in ordinates are computed, allowing for dependence between curves where appropriate [39,40]. A multiple comparison approach to testing is adopted [41,42], with the null defined as curves being indistinguishable. This is tested against both dominance and crossing of curves [43]. The null is rejected in favor of dominance if there is at least one significant difference between the ordinates of two curves in one direction and no significant difference in the other direction across 19 evenly spaced quintile points from 0.05 to 0.95. The null is rejected in favor of crossing if there is at least one significant difference in each direction [44].

## Results

Table 3 displays income quintile distributions of income and sources of health care payments in two years (years 2002 and 2007) and two types of regions (urban and rural areas), including the concentration index, the Gini coefficient and the Kakwani index of health care payment. In addition, the table displays the differences of Kakwani values in different areas and times.

**Table 3:** Distribution of household income and health care payments by income quintiles, concentration index (CI), and Kakwani index

**Year**	**Area**	**Income quintiles**	**Household income**	**General taxes**	**Public health insurance**^**a**^	**Private health insurance**	**OOP**	**Overall**
**2002**	**Urban (A)**	Q1 Poorest	5.51%	5.58%	1.94%	1.39%	4.92%	
		Q2	9.78%	9.90%	4.70%	3.37%	8.19%	
		Q3	14.74%	14.77%	14.42%	15.14%	13.71%	
		Q4	21.74%	21.64%	30.87%	27.06%	20.62%	
		Q5 Richest	48.23%	48.11%	48.08%	53.04%	52.56%	
		Gini/CI (SE)	0.4256^*^ (0.0112)	0.4232^*^ (0.0113)	0.4998^*^ (0.0263)	0.5110 (0.0597)	0.4711^*^ (0.0317)	
		Kakwani	-	−0.0024^**^ (0.0005)	0.0742^*^ (0.0297)	0.0854 (0.0613)	0.0455^*^ (0.0286)	0.0431
		(SE)						
		Weight		0.1722	0.1716	0.0229	0.6333	1
		Dominance test		D+	X	D-	None	
	**Rural (B)**	Q1 Poorest	5.59%	5.85%	10.40%	8.34%	5.51%	
		Q2	10.25%	10.97%	7.62%	2.43%	9.07%	
		Q3	14.29%	15.39%	10.69%	5.74%	11.17%	
		Q4	18.39%	18.90%	23.98%	21.62%	18.67%	
		Q5 Richest	51.48%	48.88%	47.31%	61.87%	55.57%	
		Gini/CI (SE)	0.4541^*^ (0.0446)	0.4260^*^ (0.0420)	0.3926 (0.1322)	0.5350 (0.0985)	0.4989^*^ (0.0495)	
		Kakwani	-	−0.0281^**^ (0.0060)	−0.0615 (0.1397)	0.0810 (0.1085)	0.0448^*^ (0.0295)	0.0148
		(SE)						
		Weight		0.1722	0.1716	0.0229	0.6333	1
		Dominance test		D+	None	None	None	
**2007**	**Urban (C)**	Q1 Poorest	7.42%	8.11%	3.84%	5.68%	5.90%	
		Q2	12.14%	12.40%	10.34%	7.18%	11.51%	
		Q3	16.98%	17.14%	17.49%	22.57%	16.24%	
		Q4	23.69%	23.75%	25.53%	33.79%	23.05%	
		Q5 Richest	39.77%	38.60%	42.79%	30.78%	43.30%	
		Gini/CI (SE)	0.3256^**^ (0.0044)	0.3078^**^ (0.0044)	0.3917^*^ (0.0105)	0.3345 (0.0598)	0.3743^*^ (0.0270)	
		Kakwani	-	−0.0177^**^ (0.0039)	0.0661^*^ (0.0107)	0.0089 (0.0598)	0.0488^*^ (0.0249)	0.0351
		(SE)						
		Weight		0.2453	0.2339	0.0365	0.4843	1
		Dominance test		D+	D-	None	None	
	**Rural (D)**	Q1 Poorest	7.40%	7.68%	13.37%	1.08%	6.76%	
		Q2	12.17%	12.54%	14.89%	4.68%	10.87%	
		Q3	16.29%	16.32%	17.40%	10.02%	17.31%	
		Q4	22.50%	22.33%	22.63%	25.14%	24.57%	
		Q5 Richest	41.64%	41.13%	31.71%	59.08%	40.49%	
		Gini/CI (SE)	0.3402^**^ (0.0066)	0.3305^**^ (0.0069)	0.1966^*^ (0.0394)	0.5936 (0.0673)	0.3488^*^ (0.0234)	
		Kakwani	-	−0.0097^**^ (0.0036)	−0.1436^*^ (0.0394)	0.2534 (0.0672)	0.0086^*^ (0.0223)	−0.0226
		(SE)						
		Weight		0.2453	0.2339	0.0365	0.4843	1
		Dominance test		D+	D+	D-	None	
**Inequality difference**	**Δ(urban–rural)**	2002 (A-B)	-	0.0257	0.1357	0.0045	0.0007	0.0283
		Dominance test		None	None	None	None	
		2007 (C-D)	-	−0.0080	0.2097	−0.2445	0.0402	0.0576
		Dominance test		None	D-	None	None	
	**Δ(2007–2002)**	Urban (C-A)	-	−0.0153	−0.0081	−0.0765	0.0033	−0.0080
		Dominance test		D+	D+	None	D+	
		Rural (D-B)	-	0.0184	−0.0821	0.1724	−0.0362	−0.0374
		Dominance test		D+	D+	None	None	

^*^Significant at 0.05.

^**^Significant at 0.01.

a Public health insurance in urban areas refers exclusively to BMI, and refers solely to NCMS in rural areas.

X indicates rejection of the null hypothesis that curves are indistinguishable in favor of curves crossing at the 5 percent significance level.

None indicates failure to reject the null hypothesis that curves are indistinguishable at the 5 percent significance level.

+/− indicates concentration curve dominates (is dominated by) the Lorenz curve or concentration curve in one year or area and dominates (is dominated by) the other in another year or area.

In both 2002 and 2007, the values of concentration indices were all positive, suggesting that the rich contributed a greater proportion of the health care payment than the poor. In 2002, in both urban and rural regions, almost all the values were higher than 0.4 except for the rural public health insurance (0.3926). Compared to the year 2002, all values in 2007 were nearly lower than 0.4 except for rural private health insurance (0.5936), especially the rural public health insurance (0.1966) that kept a much lower level. Three findings were discovered on the basis of the results of concentration indices. First, it was the better-off who financed a greater proportion of health care resources in China’s health care system. Second, the values of the concentration indices in rural public health insurance in both 2002 and 2007 were typically lower than the values of other health care financing channels at the same period. Third, the average level of the concentration indices in 2002 was higher than that in 2007, indicating that more financing burdens have been allocated to the low- and middle- income individuals in the more recent year, although the Gini coefficient has also decreased, which implies that the rich-poor gap is narrowing.

However, the concentration index is not a standard to judge financing equity but just a measurement to assess which population accounts for a higher share of health care resources in absolute terms. In order to evaluate the financing distribution, the Kakwani index is employed to estimate the degree of equity in health care financing system.

**Figure 2:**
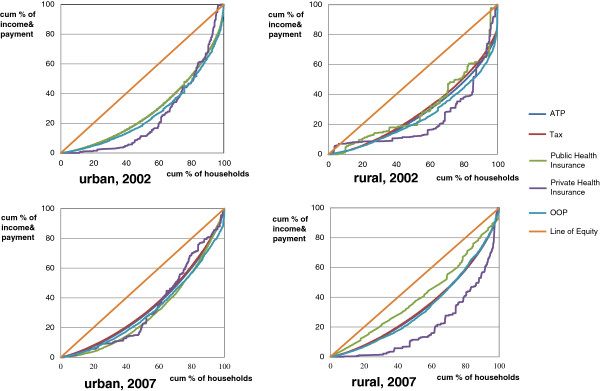
Concentration curve of health care payments and income.

In 2002, in both cities and villages, tax finance was regressive, while the finances of private health insurance and OOP payments were progressive. Unlike the progressivity consistency in urban and rural areas, public health insurance presented opposite results in cities and villages. In health care financing, insurance was progressive in urban areas but regressive in rural areas. The results indicate that, in both cities and villages, the health care financing channels of private health insurance and OOP payments were equitable, whereas the financing channel of taxes was not. In addition, fairness can be reflected by the urban public health insurance, namely the BMI. Nevertheless, it was not equitable in the rural public health insurance, CMS. Generally, the overall Kakwani index was 0.0431 in urban and 0.0148 in rural areas in 2002, while, in 2007, it was 0.0351 in urban and −0.0226 in rural areas. The classification of health care progressivity stayed the same in 2007. These results are observed directly in Figure 2, where the Lorenz curve of income and the concentration curve are plotted, giving a visual sense of the progressivity of health care payments.

Based on the results of the dominance test, the concentration curve of taxes dominated the Lorenz curve in each year and area, indicating that the financing was not equal, for the poor contributed a larger share of financing than the better off through taxes, compared with the living standard. With regard to public health insurance, compared to household expenditure, the concentration curve of the BMI crossed with the Lorenz curve in 2002, indicating that the middle class contributed a greater share of financing than the other income groups. However, the concentration curve of CMS was indistinguishable from the Lorenz curve in 2002. In 2007, the concentration curve of BMI showed a reduction in inequality, whereas the concentration curve of NCMS represented an increase in inequality. It is interesting to note that the concentration curves of OOP payments were all indistinguishable, implying that the OOP payments were not inequality-reducing and were highly correlated with the living standard.

Although the progressivity kept parallel levels between 2002 and 2007, improvement and setback could be found in the health care financing system. The differences of values of the Kakwani indices by regions and times are shown in Table 3. First, a comparison of the difference between urban and rural regions Showed that in the year 2002 (row A-B), the difference in tax financing (0.0257) was positive, while the differences in public health insurance (0.1357), private health insurance (0.0045) and OOP (0.0007) finance were positive, although the difference of the OOP Kakwani index almost equaled zero. In other words, whatever the progressivity of health care financing, compared to the rural areas, urban areas had better performance. This finding was also demonstrated by the difference (0.0283) in total progressivity in the health care financing system between urban and rural areas. Similarly, in 2007 (row C-D), the financing performance of taxes and private health insurance in urban areas was inferior to that in rural areas, especially the channel of private health insurance for the higher gap between urban and rural areas. On the contrary, public health insurance and OOP payments did better in urban areas than rural areas. In short, the difference in overall Kakwani index was 0.0576 between urban and rural areas in 2007. The dominance test showed that differences between urban and rural concentration curves were basically indistinguishable except for public health insurance in 2007, where the NCMS dominated BMI. It indicated that the BMI was more inequality-reducing than the NCMS. Second, a comparison of the difference between 2002 and 2007 showed that in urban areas (row C-A), the differences concerning taxes (−0.0153) and public and private health insurance (−0.0081 and −0.0765) were negative, indicating that health care finances have retrogressed in recent years. Conversely, the OOP payments have been making progress. Likewise, in rural areas (row D-B), taxes and private health care finances made progress while the public health care and OOP finances did not. However, the total difference between 2002 and 2007 was −0.0080 in urban areas and −0.0374 rural areas. The dominance test showed that the concentration curves in 2007 mainly dominated those in 2002, which indicated that the poor contributed more to financing the burden than the rich over the period.

## Discussion

Did China’s health care reform work in terms of the equity of health care financing? The short answer is that this is still under way. The health care financing by taxes, where the value of the Kakwani indices are close to zero but negative, was slightly regressive in both urban and rural areas in 2002 and 2007. This suggests that the tax burden for health care funding was slightly concentrated on the poor. Generally, it is universally acknowledged that tax finance is a progressive channel to fund health care, in both high and middle-income countries [10]. Compared to developed countries, where direct taxes account for the majority, the dominant part of general taxes are the indirect taxes in China, which is a pro-rich policy so that the better-off can transfer the tax burden to the poor: in 2010, the value-added tax (VAT), sales tax, and excise tax on specific goods such as alcohol, tobacco, gasoline accounted for 52.35% [45]. High reliance on indirect taxes leads to a regressive pattern through tax funding in the Chinese health care financing system. Still, tax finance has made progress in rural areas from 2002 to 2007 since an upturn of financing progressivity was noted (Table 3, row D-B). This tendency was attributed to tax renovation by China’s authorities, who have lowered the agricultural tax rate and abolished some types of taxes in rural areas since 2005. The taxpayers are peasants, most of whom are classified into the low-income group. It is suggested that, for some indirect taxes where vulnerable individuals bear more burden relative to their ability to pay, tax reduction and abolition should be done to cope with this financing inequity, especially given the context that more financing liabilities have been transferred to the low-income population in recent years.

The progressivity of public health insurance differed in urban and rural areas. In cities, BMI was progressive, while CMS (or NCMS) was regressive in villages. Besides, the values of the Kakwani indices declined in both urban and rural areas from 2002 to 2007. Evidence has shown that public health insurance can be progressive or regressive [46,47], and progressivity tends to be decreased at the early stage in the transition to universal coverage [5]. As mentioned above, China’s health sector reform took the coverage expansion seriously and covered the uninsured with the current or new insurance program. This led to the poor making more of a contribution to public health insurance than they used to, and consequently the value of the Kakwani index was decreased. However, this is a secondary reason for the decreased value of the Kakwani index. The more significant reason for divergence between urban and rural public insurance was the premium-setting policy. Both the previous CMS and the current NCMS, have one thing in common: the insured individuals are required to pay the same premium in absolute terms, regardless of their ability-to-pay. That is to say, *earns less, pays equal.* This resulted in an inequitable financing outcome for the NCMS. By contrast, persons who are covered in the BMI are required to pay a certain proportion of their earnings as premiums. In other words, *earns more, pays more*.

The OOP health care payment deserves more attention, not only because it accounts for the highest proportion of total health care financing in China, but also because it is a post-paid payment so that the identification of its financing equity is unique compared to other pre-paid payments. In some high-income countries or nations with prominent social health care financing systems, the poor households provide a larger share of financing resources with OOP payment. By contrast, in many low and middle economies, OOP payments are progressive to ability-to-pay, indicating that the better-off finance more of the funds [10]. In the common view, developing countries tend to be more equitable than developed ones in terms of OOP payment. However, as a post-paid mechanism of payment, it adheres to the law of *who pays, who gets*. In some developing countries, OOP payment is not a challenge for the high-income group in their consumption of medical goods or services, even for higher quality care at higher prices, whereas the low- and middle- income groups cannot afford to seek medical service, even the most basic medical treatment [48-51]. In our study, the OOP payments were progressive in all situations. However, unlike the progressive prepayment through taxation and public health insurance which could shift funds from the rich to the poor, progressive direct payment improved access for the wealthy who financed healthcare for themselves. Furthermore, we can see that urban areas had higher progressivity than rural ones in both 2002 and 2007, indicating the better-off in cities accounted for more health resources, especially in 2007. On the other hand, from 2002 to 2007, the progressive tendency continued in cities but not in villages, which indicated that the rural population had a higher financing equity relative to the ability-to-pay than the urban population in direct payment. In aggregate, equity evaluation on OOP payment is not judged by health care financing distribution alone but is associated with health care utilization as well.

Financing equity is just one side of health care equity and does not reflect the side of utilization. The analysis of health care utilization considers whether the better-off not only pay more but also receive more health care. With pre-paid payment, we can infer whether the poor enjoy more services or not since they are more inclined to make a greater contribution relative to their ability to pay. Different from the financing equity that is assessed by the Kakwani index, the equity of utilization is judged by the concentration index directly since an individual’s need for health care utilization has nothing to do with income [52]. The distribution of health care utilization in Gansu province is estimated in Table 4.

**Table 4:** Concentration indices for health care utilization in Gansu, China, 2002 and 2007

**Year**	**Area**	**Hospital outpatient care**	**Hospital inpatient care**
**2002**	**urban**	−0.0047^*^	0.1359
		(0.0365)	(0.0533)
	**rural**	0.2230^*^	0.3625
		(0.0326)	(0.0666)
**2007**	**urban**	0.1451	0.3281
		(0.0502)	(0.0504)
	**rural**	0.0564^*^	0.3310^*^
		(0.0358)	(0.0460)

^*^Significant at 0.05.

In 2002, almost all the values of concentration indices for hospital care were positive, indicating that the rich received a larger share of health care resources. Similarly in 2007, the better-off absorbed a higher proportion of care than the poor. It was suggested that for each category of hospital care, there was basically a pro-rich bias, which is consistent with our finding that OOP payment was heavily relied upon by the high-income group in Gansu province. In other word, the rich paid more and receive more while the poor received less because they simply could not afford medical expenses and so sought no treatment. From 2002 to 2007, for the increase of concentration index for each category of urban hospital care, we tentatively put forward that OOP payment played a dominant role in health care payment and that other prepayments such as public health insurance could not perform as well in improving health care utilization. On the contrary, the concentration index for each category of rural hospital care has fallen in recent years, which is attributed to the introduction of NCMS, and more importantly, lower coinsurance and more covered services that lead to the villagers having more access to health care, especially to outpatient care at lower prices. We believe that a health insurance (e.g. BMI), even if it is financing equitable, would not improve the extent of use of health care, especially for the low-income group, if the policy’s goal exclusively focused on the coverage rather than the benefit package, such as lower copayment and deductibles, more insured health care services, and so on.

However, our study primarily used data primarily prior to the year 2007. China’s health care reform after 2007 has been affected by the *CNY 850 billion Plan* for the health sector. Benefited by the additional government spending, the average premiums of NCMS and URBMI have increased from 58.9 RMB in 2007 to 230 RMB in 2011, and 100 RMB in 2007 to 300 RMB in 2011 [26], respectively. More significantly, individual contributions to premiums stayed the same over the period. The increase of premium was financed exclusively from the central and state governments. Put differently, this has narrowed the gap between the rich and the poor for the individual’s proportion of the total premium. In addition, more coverage for ambulatory care and further co-payment reductions in priority diseases in the inpatient service improves patient access to health care, especially for the poor. Both measures might reduce inequality in health financing and utilization.

Some limitations of our data must however be acknowledged, because they may affect the interpretation of our results and call for caution in their generalization as a basis for informing the policy debate about healthcare reforms in China.

One major limitation in our study is that it examined one single province of China. It is not likely to fully represent national health care financing characteristics. Notwithstanding this limitation, our study employed percentages and indices to evaluate the implementation of national policies and programs in a whole population. Because of this feature, it is less linked with the provincial economic level and its geographic location. To some extent, a sub-national financing equity can reflect the national financing distribution. We look forward to cross-province comparisons of financing equity to be conducted in the future. Although regional economic development has been taken into consideration in our research through using the Kakwani index, we cannot say with certainty that the renovations implemented in the health care financing system, for example, the expansions of NCMS and BMI, have led to the observed changes in progressivity. Other factors that are difficult to control in our study, such as changes of geographic access to health care, patient satisfaction, and awareness of prevention, would influence financing progressivity. This may be avoided in future studies, should another similar socioeconomic province without similar health policy interventions be collected as the control group, using a difference-in-differences approach.

A limitation in data collection is that we used the financing distribution of indirect taxes to represent general tax distribution, owing to the inaccessibility of data collection for direct taxes in the household health interview surveys. Although indirect taxes constitute the major part of general taxes in China, it should be noted that progressivity of tax financing might be underestimated in our study, considering the progressive effect of direct taxes. In addition, a proxy for living standards was household expenditures (per adult equivalence), which is likely to understate the living standards of rural residents, for household production cannot be neglected in these areas.

Last, our study focused on the period between 2002 and 2007. However, as discussed above, policy implemented after 2007 has affected health care financing already. A new round of household survey and analysis would explore the latest financing pattern in health care reform in further studies.

## Conclusions

The theme of China’s health sector in the first decade of 21^st^ century is the struggle for health care reform. Based on our empirical study, we found that more low- and middle-income households have begun to undertake a higher proportion of the health care financing burden compared to the past. On reason is that the economic gap between the rich and the poor has narrowed. Another reason is the outcomes of the measures launched by renovations in the health sector. In health care financing reform, general taxes are slightly pro-rich since the indirect taxes account for the majority and are preferred by the better-off. Based on a successful experience in rural areas, some small-scale tax exemption, especially for those taxes that concentrate on vulnerable groups, will play a large part in equity improvement.

Both urban and rural health insurance have two sides: the Basic Medical Insurance (BMI) does well in financing equity but not for utilization; the New Rural Cooperative Medical Scheme (NCMS) is not equitable in financing, but the poor have more access to health services. Results indicate that, by financing standards, BMI in cities is evenly distributed while NCMS is not. However, the equity performance is partially associated with the coverage. Key for health care reform is not only increasing the number of the insured population but also optimizing the benefit packages’ to broaden access to medical care for more persons, especially for poor individuals. This also explains why the rural areas had better utilization equity than urban areas. Despite their flaws, BMI and NCMS did improve financing equity and health utilization, respectively. However, OOP payment still dominated as the major part of total financing in the year 2007 (Table 1), which limits the role played by the prepayment systems, which account for a minor part of health care financing. These results should inform strategies to adjust financing policies and mechanisms under the current Chinese health care reform effort.

## Abbreviations

ATP: Ability-to-pay; GWIS: Government Welfare Insurance Scheme; LIS: Labor Insurance Scheme; OOP: Out-of-pocket; TEH: Total expenditure on health; VAT: Value-added tax; BMI: Basic Medical Insurance; UWBMI: Urban Workers Basic Medical Insurance; URBMI: Urban Resident Basic Medical Insurance scheme; CMS: Cooperative Medical System; NCMS: New Rural Cooperative Medical Scheme.

## Competing interests

The authors declare that they have no competing interests.

## Authors’ contributions

Prof Yuxin Zhao led the study. She designed the study, led the data collection, analysis, and interpretation. Mingsheng Chen contributed to the study design, provided input into the data analysis, and wrote the first draft of the manuscript. Prof Wen Chen contributed to the study design, reviewed the manuscript and helped the writing of the final draft manuscript. All authors read and approved the final manuscript.

## Pre-publication history

The pre-publication history for this paper can be accessed here:

http://www.biomedcentral.com/1472-6963/12/466/prepub
